# Eco-production of silica from sugarcane bagasse ash for use as a photochromic pigment filler

**DOI:** 10.1038/s41598-020-66885-y

**Published:** 2020-06-18

**Authors:** Prinya Chindaprasirt, Ubolluk Rattanasak

**Affiliations:** 1 Sustainable Infrastructure Research and Development Center, Department of Civil Engineering, Faculty of Engineering, Khon Kaen University, Khon Kaen, 40002 Thailand; 2 Academy of Science, The Royal Society of Thailand, Office of The Royal Society, Dusit, Bangkok, 10300 Thailand; 3 Department of Chemistry, Faculty of Science, Burapha University, Chonburi, 20131 Thailand

**Keywords:** Environmental chemistry, Green chemistry

## Abstract

Sugarcane bagasse is a significant renewable energy source for the sugar and bioethanol industries. Bagasse ash is the waste from the combustion process and is mostly disposed of as landfill. Only a small quantity of bagasse ash is utilized as pozzolan in concrete, and a considerable quantity is left unused due to its high carbon and crystallite content. Generally, bagasse ash is rich in silica (SiO_2_), and it is thus an alternative source for silica extraction. In this paper, a low-energy and low-chemical consumption method is proposed to obtain silica from bagasse ash using alkali extraction and acid precipitation. The physical and chemical properties of the extracted silica are described. A silica yield of 80% and moisture absorption of 73% were achieved. The silica had amorphous phases and was light gray in color owing to the presence of carbon from incomplete combustion. Bagasse silica was used as an extender filler in an expensive photochromic pigment to increase the bulk volume. It was found that a pigment-to-silica mass ratio up to 1:10 could be used for thick-layer painting. However, a ratio of up to 1:3 is recommended for thin-layer screen-printing on fabrics. The bagasse ash silica-pigment blends have very good color fastness when washing; however, frequent aggressive washing should be avoided.

## Introduction

Sugar and bioethanol manufacturing processes produce a large quantity of sugarcane bagasse, which is used as a renewable energy source. In 2017, 28.1 million metric tons of bagasse was generated in Thailand, and this was mainly used in the factories as an alternative energy source for steam generation and biomass power plants^1^. Waste from the combustion process in the form of ash amounts to approximately 20%wt of bagasse. Most of the bagasse ash is disposed of as landfill. The chemical composition of bagasse ash depends on the burning condition, i.e., burning temperature, burning duration and air intake. Owing to the incomplete burning of bagasse, carbon is also found in bagasse ash. Generally, bagasse ash has a high silica (SiO_2_) content. Silica content is influenced by the availability of silicon in soil; sugarcane roots play a significant role in the absorption of silicic acid from the soil and transporting it toward the shoots, where it is deposited as amorphous silica. The transpiration process in the plants enhances silica deposition in all parts by water transmission^2^. However, the amount of silica in bagasse varies depending on the species and maturity of the sugarcane, geological and soil conditions, fertilizer used, and cultivation practice. Silica forms in bagasse ash consist of amorphous phases and crystalline phases (quartz and cristobalite). It has been reported that quartz could also come from sand stuck to the sugarcane during harvest. Cristobalite is a crystal morphology of silica resulting from the high combustion temperature of bagasse^3,4^. Researchers have reported that bagasse ash has the potential for use as pozzolan in concretes since it contains amorphous phases that can react with chemical compounds in a cement mixture^5,6^. Bagasse ash needs to be ground to increase its reactivity through increased fineness and surface area. However, the incorporation of a high volume of bagasse ash adversely affects the binding and physical properties of pozzolanic cement, and so small quantities of bagasse ash are used. Bagasse ash contains high levels of SiO_2_, similar to rice husk ash, and it is used as a source material for the extraction of SiO_2_.

Generally, the commercial production of silica gel involves the acidification of sodium silicate solution from melting quartz sand with soda ash (sodium carbonate, Na_2_CO_3_) at a high temperature of 1300^°^C^7^. This process consumes a lot of energy. A simple method with low-energy consumption has been proposed for extracting SiO_2_ from rice husk ash (RHA)^8^. The method involves boiling the rice husk ash in a low concentration of NaOH to form a sodium silicate solution; silica is precipitated in the acid solution. The silica particles are used in many applications, such as a reinforcing agent in rubber, cleaning agent in toothpaste, filler in paint, and dye removal^9^.

The high content of silica in bagasse ash makes it an attractive alternative silica source for silica extraction. Several studies have proposed the syntheses of silica from bagasse ash. However, the application of silica extraction from bagasse ash has rarely been reported^2,10–13^. Therefore, this paper presents the synthesis of silica from bagasse ash by an alkali extraction and acid precipitation method that consumes low levels of both energy and chemicals. Photochromic pigment is a specially designed powder which changes in color under direct sunlight and is very expensive, even in small quantities. Photochromic pigment is generally used for screen-printing, automotive paint, and fabric paint. In this research, the use of silica from bagasse ash as an extender filler in photochromic pigment is also investigated as a means of increasing bulk volume and reducing cost.

## Materials and Methods

### Materials

Bagasse fly ash was collected from a local sugar factory in Thailand. As-received ash was used to extract silica. The microstructure of bagasse ash was studied using a scanning electron microscope (SEM, Leo 1450VP, gold coating). Oxide compounds were analyzed by X-ray fluorescence spectrometry (XRF, Rigaku ZSX Primus). Loss on ignition (LOI) was calculated from the weight loss of the ash heated at 950 ± 50 °C for 15 min according to ASTM C114-15*: Standard test methods for chemical analysis of hydraulic cement*. A morphological study was carried out using X-ray diffraction spectroscopy (XRD, Rigaku MiniFlex).

1M hydrochloric solution (HCl) and 2.5 M HCl were used as the acid solutions for ash pre-treatment and silica precipitation, respectively. 1 M sodium hydroxide solution (NaOH) was prepared for the extraction of silica from bagasse ash. In addition, photochromic reversible pigment (white to magenta) and basecoat binder were purchased from international suppliers.

### Experimental methods

#### Bagasse ash pre-treatment

To remove the oxide compounds of minor elements from bagasse ash, an acid treatment was performed. 100 g of as-received bagasse ash was suspended in 600 ml 1 M HCl and stirred continuously by magnetic stirrer in a controlled 25 °C room for 2 hours. The ash was then filtered and washed with hot water until the pH of the filtrate was 7. Subsequently, the pre-treated ash was dried in an oven at 100 ± 5 °C for 5 hours.

#### Silica extraction

The acid-treated bagasse ash was dispersed in 1 M NaOH in a beaker with ash-to-base ratios of 1:5, 1:8 and 1:10 w/v. The mixture was heated at 90 ± 5 °C for 1 hour and stirred by a magnetic stirrer. The beaker was covered with watch glass during the heating process, after which, the mixture was left to cool to room temperature. The ash residue was filtered off, and the filter cake was washed with hot water (double the volume of NaOH used). The filtrate, i.e. sodium silicate and washing water, was adjusted to pH of 7 with 2.5 M HCl and left for silica aging at room temperature for 24 hours. The silica was then filtrated and washed with hot water and dried at 100 ± 5 °C for 5 hours. The percentage yield of silica was calculated based on the silica content of the bagasse used.

#### Silica characterization

The morphology of the obtained silica was characterized by XRD and attenuated total reflectance-Fourier transform infrared spectroscopy (ATR-FTIR, Perkin Elmer Frontier) and compared with commercial silica gel. The silica was ground and sieved through sieve no. 10 (2.00 mm opening) and retained on sieve no. 20 (0.84 mm opening) for use in the moisture absorption test. The silica was oven-dried at 100 ± 5 °C for 1 hour and then cooled to room temperature. The moisture absorption of the sample was then measured in accordance with the requirement of ASTM D 570: *Standard test method for water absorption of plastics*. The weight change of the sample was recorded, and the percentage of moisture absorption was calculated based on the weight of the dried sample^14^.

#### Application of silica as an extender filler

The silica extracted from bagasse ash was used as an extender filler to increase the volume of photochromic reversible pigment, which is a water-based powder. The silica was ground and sieved to obtain an 85% portion through sieve no. 345 (45 μm) and retained on sieve no. 200 (74 μm). The silica was then blended with photochromic reversible pigment at pigment-to-silica mass ratios of 1:0, 1:1, 1:3, 1:5, 1:8 and 1:10. The blends were dissolved in water and binder as recommended by the supplier to improve durability. The blended paste was painted in a thick layer on 200-gram drawing paper. The paper was dried at room temperature (25 °C), and color imaging was performed under direct sunlight. Imaging color was then converted into an 8-bit grayscale color resulting in 256 different intensity levels. The ImageJ program was used to identify the gray shade, where 0 means black color and 256 means white color.

In addition, fabric screen-printing was performed on calico cotton fabric using a plain mesh print screen. Then, the printed fabrics were heat cured at 120 °C for 5 min in a hot air oven to improve durability. A test of color fastness when washing was performed on the fabrics^15^. This test reported the photochromic color build-up before and after the wash fastness test.

## Results and Discussion

### Characteristics of bagasse ash

**Figure 1:**
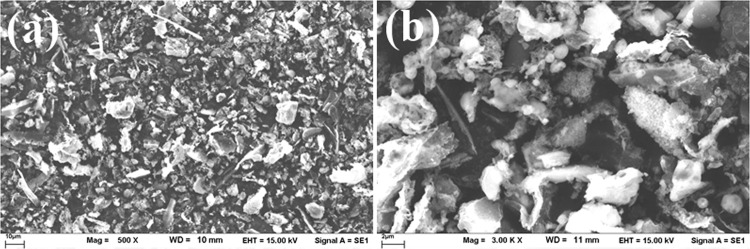
Microstructure of bagasse ash: (**a**) 500x (**b**) 3000x.

The bagasse ash was black in color with a mean particle size (D50) of 13.98 µm measured by a particle size analyzer (Malvern Mastersizer S). Its specific gravity was 1.9, and the particle shape was irregular, as shown by the SEM in Fig. 1. The oxide compositions of the bagasse ash from XRF are shown in Table 1. The main oxide compositions of the as-received bagasse ash were SiO_2_ (54.9%), Fe_2_O_3_ (10.0%), Al_2_O_3_ (7.8%), and CaO (4.9%) with a high LOI value of 12.2%, indicating high carbon content as a result of incomplete burning.

**Table 1:** Oxide compositions and LOI of the bagasse ash.

Compounds	Concentration (%wt)	
	As-received bagasse ash	Acid pre-treated ash
SiO_2_	54.9	66.3
Al_2_O_3_	7.8	4.6
CaO	4.9	0.8
Fe_2_O_3_	10.0	10.4
SO_3_	1.2	0.2
MgO	2.5	1.7
K_2_O	3.0	1.2
Na_2_O	0.2	0.2
others	3.2	2.1
LOI	12.2	12.5

After the acid pre-treatment, the silica content in the ash increased and reduced amounts of metal oxides were detected. The amounts of Al_2_O_3_, CaO, SO_3_, MgO and K_2_O were significantly reduced due to the reaction with the acid. In particular, CaO dissolved easily in the acid solution. The metal elements reacted with HCl and formed chloride salt solutions, as shown in Eqs. 1–5. In addition, the LOI of the pre-treatment ash did not change greatly because the acid solution did not react with carbon.

1 $${\rm{A}}{{\rm{l}}}_{{\rm{2}}}{{\rm{O}}}_{{\rm{3}}}({\rm{s}}){\rm{+}}{\rm{6}}{\rm{H}}{\rm{C}}{\rm{l}}({\rm{a}}{\rm{q}}){\rm{\to }}{\rm{2}}{\rm{A}}{\rm{l}}{\rm{C}}{{\rm{l}}}_{{\rm{3}}}({\rm{a}}{\rm{q}}){\rm{+}}{\rm{3}}{{\rm{H}}}_{{\rm{2}}}{\rm{O}}({\rm{l}})$$

2 $${\rm{C}}{\rm{a}}{\rm{O}}({\rm{s}}){\rm{+}}{\rm{2}}{\rm{H}}{\rm{C}}{\rm{l}}({\rm{a}}{\rm{q}}){\rm{\to }}{\rm{C}}{\rm{a}}{\rm{C}}{{\rm{l}}}_{{\rm{2}}}({\rm{a}}{\rm{q}}){\rm{+}}{{\rm{H}}}_{{\rm{2}}}{\rm{O}}({\rm{l}})$$

3 $${\rm{S}}{{\rm{O}}}_{{\rm{3}}}({\rm{s}}){\rm{+}}{\rm{6}}{\rm{H}}{\rm{C}}{\rm{l}}({\rm{a}}{\rm{q}}){\rm{\to }}{\rm{S}}{\rm{C}}{{\rm{l}}}_{{\rm{6}}}({\rm{a}}{\rm{q}}){\rm{+}}{\rm{3}}{{\rm{H}}}_{{\rm{2}}}{\rm{O}}({\rm{l}})$$

4 $${\rm{M}}{\rm{g}}{\rm{O}}({\rm{s}}){\rm{+}}{\rm{2}}{\rm{H}}{\rm{C}}{\rm{l}}({\rm{a}}{\rm{q}}){\rm{\to }}{\rm{M}}{\rm{g}}{\rm{C}}{{\rm{l}}}_{{\rm{2}}}({\rm{a}}{\rm{q}}){\rm{+}}{{\rm{H}}}_{{\rm{2}}}{\rm{O}}({\rm{l}})$$

5 $${{\rm{K}}}_{{\rm{2}}}{\rm{O}}({\rm{s}}){\rm{+}}{\rm{2}}{\rm{H}}{\rm{C}}{\rm{l}}({\rm{a}}{\rm{q}}){\rm{\to }}{\rm{2}}{\rm{K}}{\rm{C}}{\rm{l}}({\rm{a}}{\rm{q}}){\rm{+}}{\rm{3}}{{\rm{H}}}_{{\rm{2}}}{\rm{O}}({\rm{l}})$$

**Figure 2:**
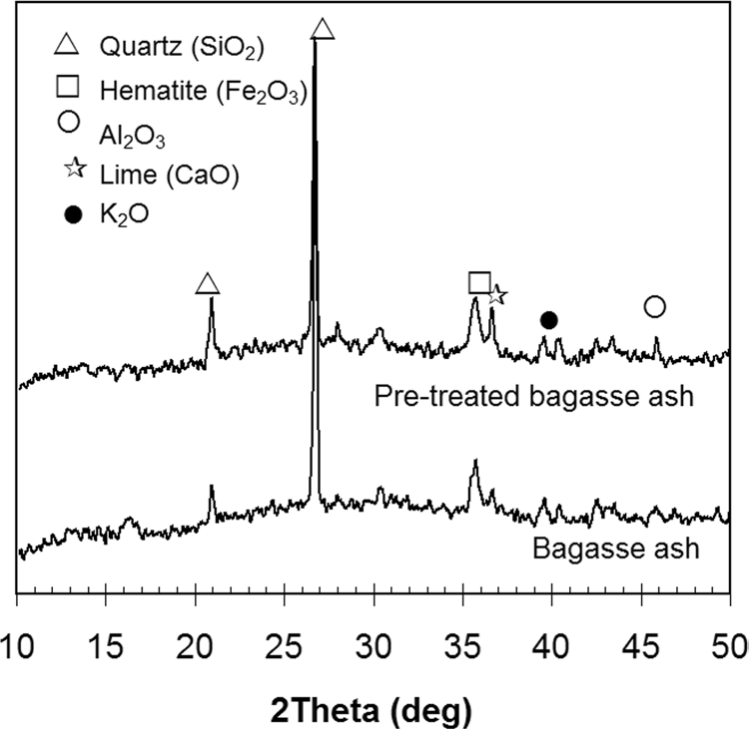
XRD patterns of the as-received bagasse ash and pre-treated bagasse ash.

The morphology of the ash was analyzed by XRD, and the results are shown in Fig. 2. The mineral compositions from diffraction peaks for SiO_2_, Fe_2_O_3_, Al_2_O_3_, and CaO, were identified using the Match program. Crystalline silica in quartz form was easily noticed with sharp peaks at 20.9 and 26.6 2θ. The broad peak of the XRD pattern showed the presence of amorphous phases. The results also showed that the acid pre-treatment did not significantly change the morphology of the bagasse ash.

### Properties of silica

When the acid-treated bagasse ash was extracted with NaOH solution at 90 ± 5 °C for silica, sodium silicate solution (Na_2_SiO_3_) was obtained, as shown in Eq. 7. The precipitation reaction of silica from Na_2_SiO_3_ with HCl acid is shown in Eq. 8.

7 $${\rm{S}}{\rm{i}}{{\rm{O}}}_{{\rm{2}}}({\rm{s}}){\rm{+}}{\rm{2}}{\rm{N}}{\rm{a}}{\rm{O}}{\rm{H}}({\rm{a}}{\rm{q}}){\rm{\to }}{\rm{N}}{{\rm{a}}}_{{\rm{2}}}{\rm{S}}{\rm{i}}{{\rm{O}}}_{{\rm{3}}}({\rm{a}}{\rm{q}}){\rm{+}}{{\rm{H}}}_{{\rm{2}}}{\rm{O}}({\rm{l}})$$

8 $${\rm{N}}{{\rm{a}}}_{{\rm{2}}}{\rm{S}}{\rm{i}}{{\rm{O}}}_{{\rm{3}}}({\rm{a}}{\rm{q}}){\rm{+}}{\rm{2}}{\rm{H}}{\rm{C}}{\rm{l}}({\rm{a}}{\rm{q}}){\rm{\to }}{\rm{S}}{\rm{i}}{{\rm{O}}}_{{\rm{2}}}({\rm{s}}){\rm{+}}{\rm{2}}{\rm{N}}{\rm{a}}{\rm{C}}{\rm{l}}({\rm{a}}{\rm{q}}){\rm{+}}{{\rm{H}}}_{{\rm{2}}}{\rm{O}}({\rm{l}})$$

It has been reported that a high concentration of NaOH (> 1 M) does not significantly improve the silica yield, and so 1 M NaOH is recommended for the extraction of silica^8,12^. Silica was extracted from black bagasse ash and dissolved into NaOH solution forming a liquid phase of sodium silicate solution. The silica started to precipitate at a pH lower than 10 from the obtained sodium silicate solution using 2.5 M HCl, as shown in Eq. 8. In the process, a pH of 7 was maintained and the gel was formed during this silica aging period. It should be noted that an increase in the HCl concentration (>2.5 M) had an adverse effect on silica yield owing to the re-dissolution of silica in a high acid environment^12^. After filtration, washing the silica with hot water helped lower the Na and K contents of the final products, and whiten the silica resulting in purer silica. Silica from this synthesis method was xerogel type with liquid phase in pores and could be removed by evaporation^8^.

The ash-to-NaOH ratio also affected the silica yield, moisture absorption and color of products, as presented in Table 2. With an ash-to-NaOH ratio of 1:5 w/v (low NaOH), the yield of silica was low due to insufficient NaOH. In addition, dense silica was formed as indicated by the low moisture absorption and yellowish color. In this case, the remaining polyvalent iron (Fe^3+^) was strongly absorbed onto the silica surface and trapped inside the silica network during gel formation in the silicate solution^7^. An increase in NaOH volume to an ash-to-NaOH ratio of 1:8 w/v resulted in higher yield and moisture absorption, and a light gray color product was obtained. However, the yield of silica decreased significantly when the high volume NaOH solution with the ratio of 1:10 w/v was applied, due to the lower concentration of the sodium silicate solution. Prolonged aging time and reduced volume of washing water were required to increase the yield. An ash-to-NaOH ratio of 1:8 w/v was, therefore, selected for the extraction of silica from bagasse ash for the next application. The silica gel production from bagasse ash by the alkali extraction method offers low chemical and energy consumption compared to the traditional method using molten quartz sand. The traditional method consumes energy of 126 watt-hours (Wh) to produce 1 kg of silica, 21 Wh of which is electrical energy and the remainder is mainly from fossil fuel^16^.

**Table 2:** Yield and moisture content of silica.

Silica	Ash: NaOH ratio (w/v) for extraction condition	Yield (%)	Moisture absorption (%)	Color
No. 1	1:5	61.2	56.7	Yellowish
No. 2	1:8	80.5	73.4	Light gray
No. 3	1:10	63.9	75.6	Light gray

**Figure 3:**
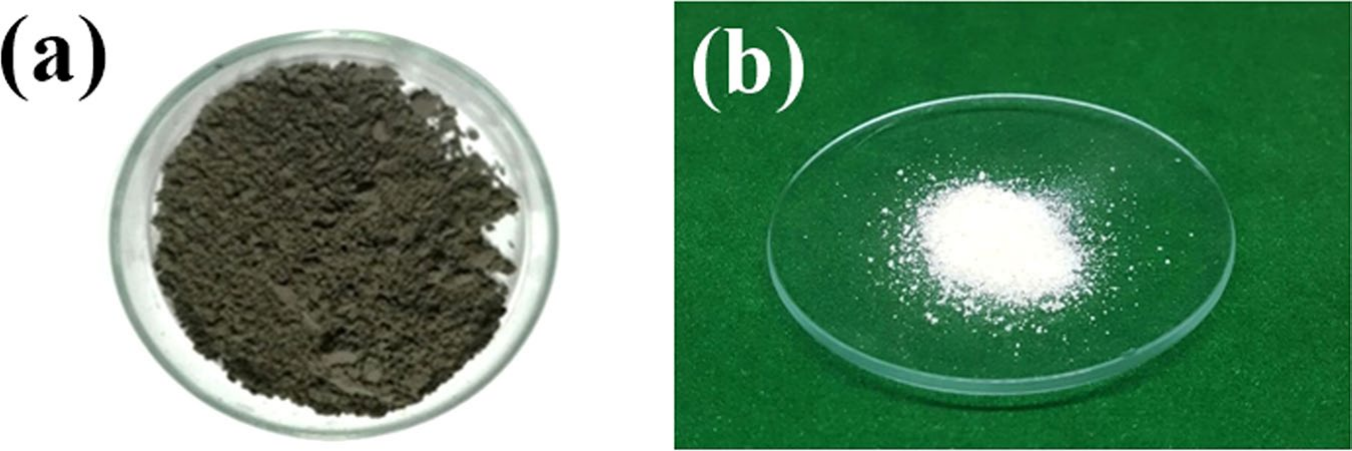
Images of (**a**) bagasse ash (**b**) silica.

Figure 3 shows the color of the bagasse ash and the silica after extraction. The silica color was light gray owing to the presence of unburnt carbon in the bagasse ash. The color of silica could, however, be made whiter with increased washing of the acid-precipitated silica with hot water.

**Figure 4:**
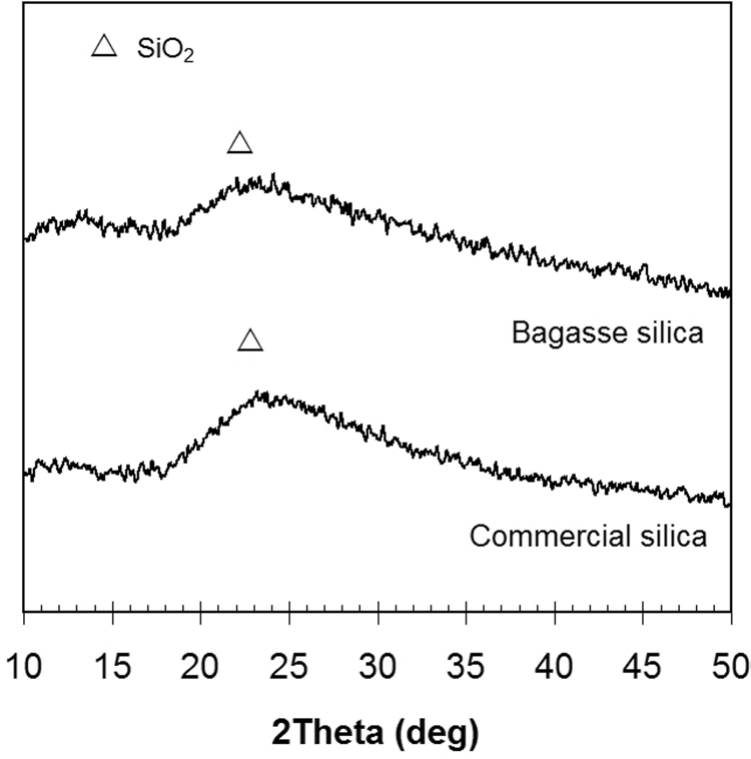
XRD patterns of bagasse silica and commercial silica.

**Figure 5:**
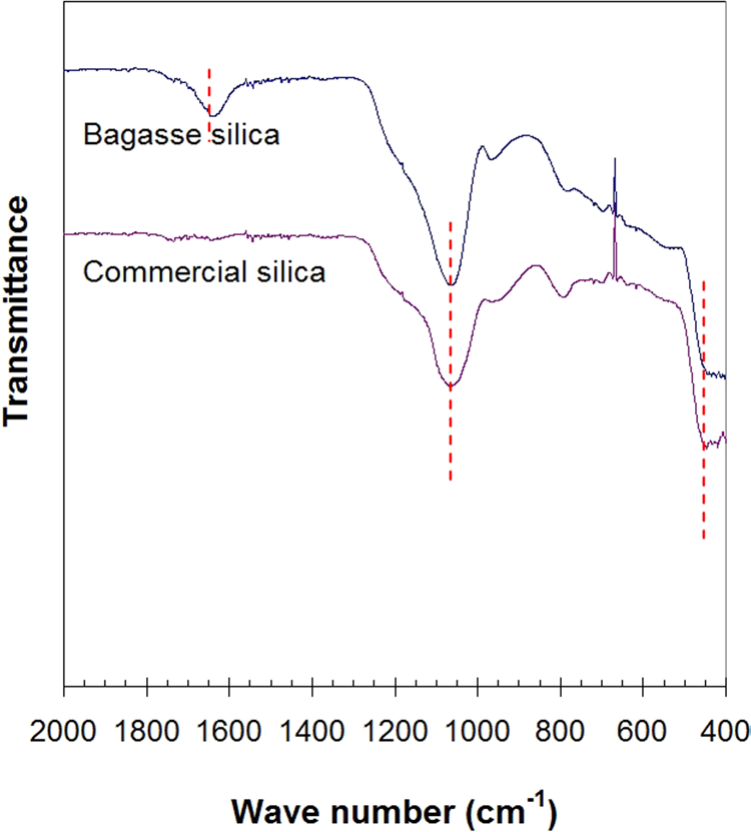
FTIR peaks of bagasse silica and commercial silica.

Figure 4 presents the XRD pattern of bagasse silica and commercial silica used as a food desiccant in snack packages. The patterns of both silicas were similar, and SiO_2_ mineral was identified at 23-24° 2θ. The bagasse silica was amorphous, as indicated by the XRD broad peak and the disordered arrangement of the silica network. For comparison, the FTIR spectra of bagasse silica and commercial silica are shown in Fig. 5. Si-O bands were observed in the spectra at wave numbers of 450 and 1050 cm^−1^ attributed to the Si-O-Si symmetric stretching vibration at 450 − 455 cm^−1^ and the Si-O-Si asymmetric stretching vibration at 1050–1070 cm^−1 11^. An absorption band at 1640 cm^−1^ was found in bagasse silica, showing the presence of a small amount of water with a bending vibration of either free -OH groups or free H_2_O molecules.

Silanol groups are formed on the silica surface during the condensation-polymerization of silicate (Si(OH)_4_). In amorphous silica, the bulk structure is a random packing of a Si(OH)_4_^4-^ unit. Three strong absorption bands measured by FTIR are generally found at 800, 1100 and 1250 cm^−1^ attributed to fundamental Si-O vibrations^17^. Silanol groups are generally found on the surface and within the structure of silica particles. The silanol surface OH groups are the main centers of water molecule absorption. The water molecule can be associated by hydrogen bonds to surface silanols and sometimes to internal silanol groups. There are two types of physically adsorbed water (physisorbed water) on the silica surface, i.e., low activation energy and high-low activation energy^18^. Therefore, for the use of silica filler in pigment, a water-based pigment is used in order to obtain fully the absorption of pigment with silanol groups on silica surface.

### Application of silica as extender filler

**Figure 6:**
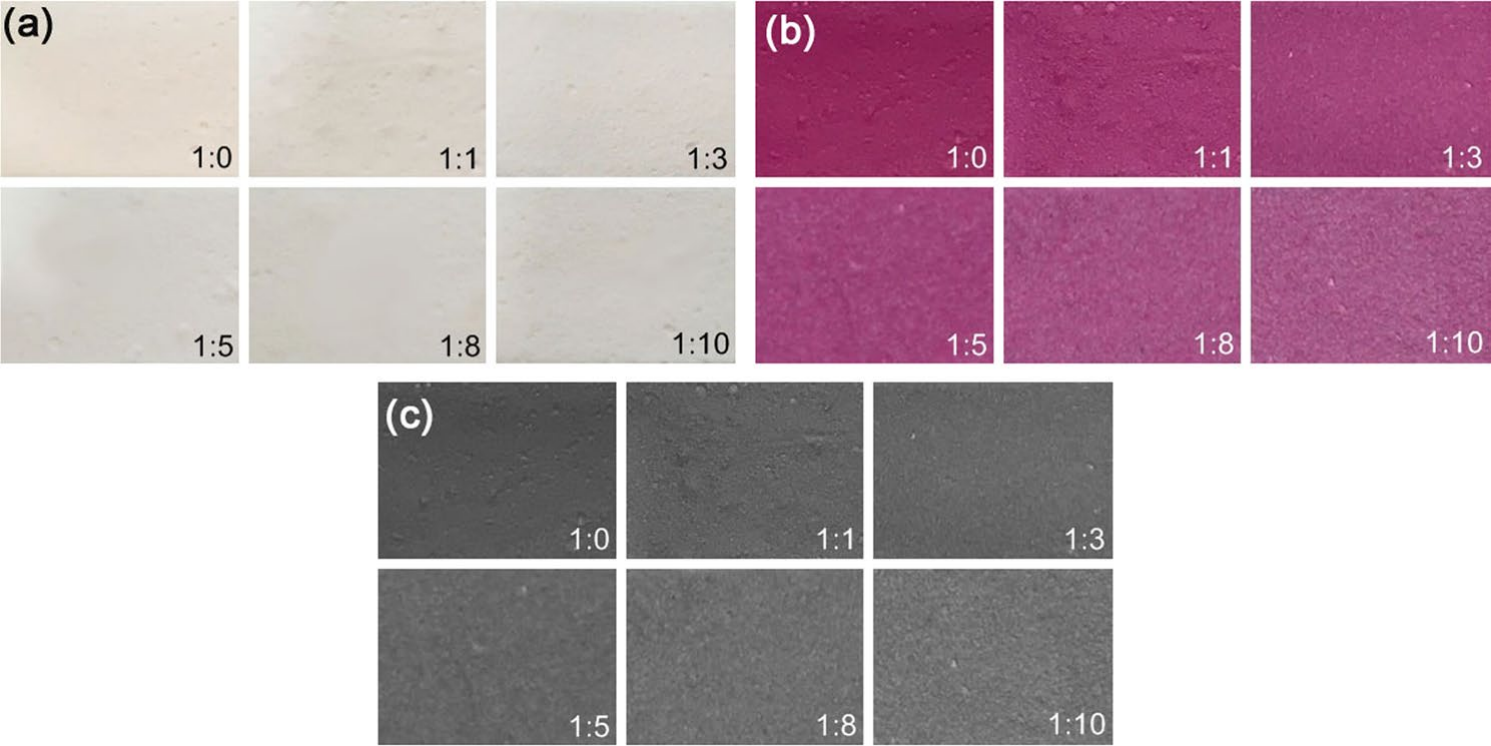
Colors of photochromic pigment blends with silica: (**a**) indoor (**b**) under sunlight (**c**) gray shade for ImageJ analysis.

The silica was mixed with the photochromic reversible pigment (white to magenta) with pigment-to-silica mass ratios of 1:0, 1:1, 1:3, 1:5, 1:8, and 1:10. The blends were then dissolved with the exact amount of water recommended by the supplier. Since the paint was water-based and dissolved in water, a soft base binder was used as a thickener, and durability was increased by heat curing at 120 °C for 5 min. The paste was obtained and painted in a thick layer on 200-gram drawing paper. The colors of blends are shown in Fig. 6. 8-bit grayscale color by the ImageJ program was used to measure the intensity levels, where 0 means black color and 256 means white color. The intensity levels are shown in Table 3.

**Table 3:** Intensity levels of gray shade of blend pigments.

Pigment-to-silica mass ratio	Gray shade	Shade difference (%)
1:0	82	0.0
1:1	91	−11.0
1:3	103	−25.6
1:5	104	−26.8
1:8	106	−29.3
1:10	107	−30.5

From the intensity levels of gray shade (Table 3), it can be seen that the blending of photochromic pigment and silica resulted in a lighter color under sunlight with a higher intensity level. A rougher surface was detected with the increase in silica content. The color intensity dropped by 11% when the silica was blended with pigment at a ratio of 1:1. Since silica has a silanol group (Si-O-H) with hydrophilic character, the silica surface thus physically adsorbs water molecules, resulting in surface absorption of the water-based photochromic pigment. Using a carrier of spherical particles with a narrow size distribution, pigments with a required covering power can be obtained^19^. In addition, silica improved the dispersibility and increased the specific volume of pigment. Since silica has silanol groups on the surface, the absorption of water molecules in the pigment mixture can be obtained^17^. The test results showed that the obtained shade differences were in the range of 25–10% at pigment-to-silica mass ratios of 1:3 to 1:10. When thick-layer painting was performed using the blended pigment, silica could, therefore, be blended with the photochromic pigment up to a ratio of 1:10. The high silica ratio led to a slightly lighter color.

**Figure 7:**
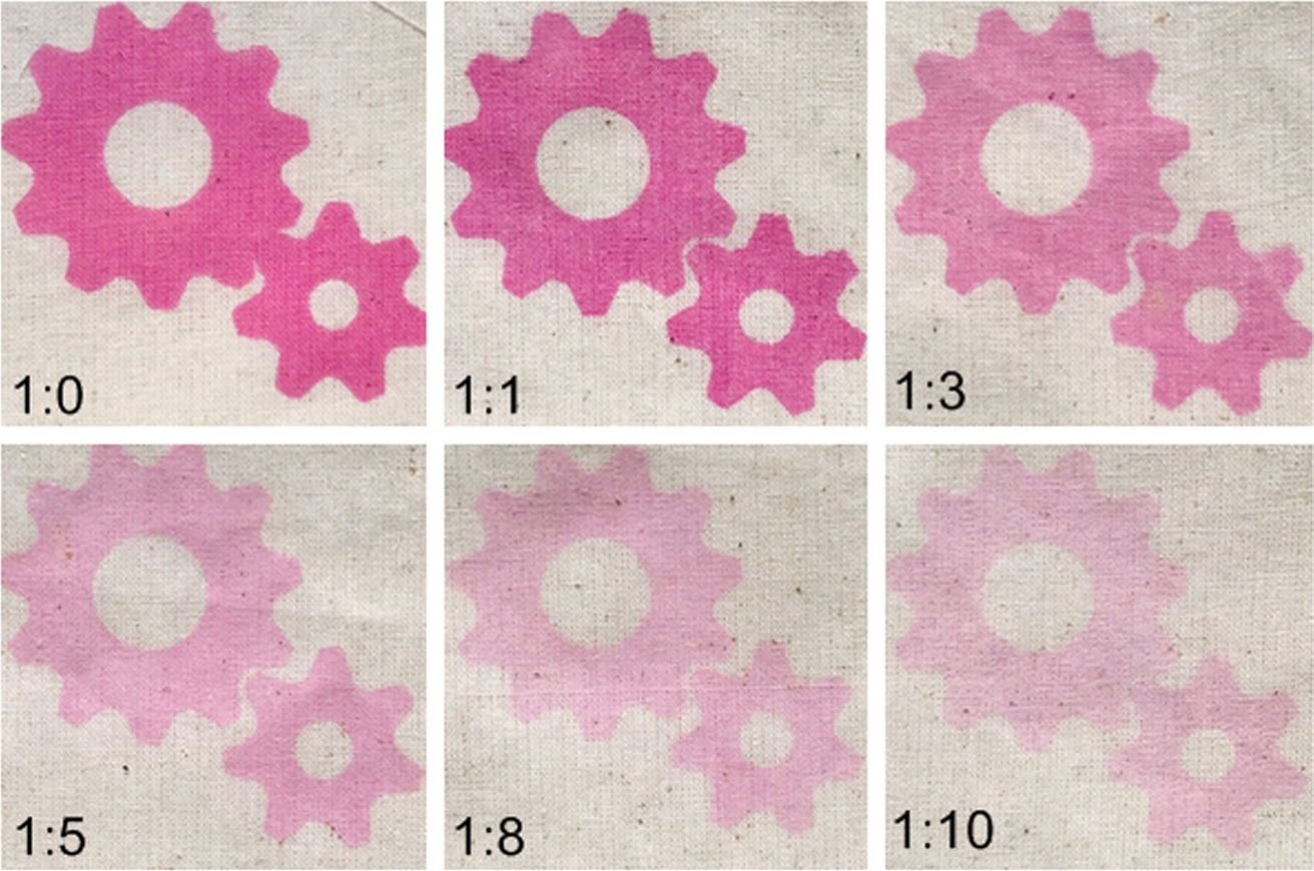
Colors of photochromic pigment blends with silica on fabrics (under sunlight).

In addition, thin-layer printing using a plain mesh print screen was performed on calico cotton fabrics. The results of various pigment-to-silica mass ratios are shown in Fig. 7. Heat curing at 120 °C for 5 min was applied to cure the soft base binder, leading to improved durability of the screen-print product. The color of the blended pigment with high silica content was significantly lighter than that of the control. For optical investigation, a pigment-to-silica mass ratio higher than 1:3 is not recommended. Color fastness to washing was studied on the fabrics. Nine pairs of grayscale color chips were used to rate the visual difference and contrast compared to unwashed fabric. On this scale, 5 indicates the best rating with no visual change, and 1 is the worst rating with a large visual change^20^. The result of color fastness to washing is presented in Table 4.

**Table 4:** Changes in shade after color fastness test on fabrics.

Pigment-to-silica mass ratio	Changes in shade		
	1^st^ wash	2^nd^ wash	3^rd^ wash
1:0	5	4–5	4–5
1:1	5	4–5	4–5
1:3	5	4–5	4–5
1:5	4-5	4–5	4
1:8	4–5	4–5	4
1:10	4–5	4–5	4

From Table 4, it can be seen that the color was lost after washing, particularly with a high volume of silica blend. It is proposed that the polymeric binder matrix loosened around the colorant molecule with initial washing, and the wash fastness properties of the photochromic prints were associated with the binder. Since most photochromic pigments are aromatic molecules, the degree of photo coloration decreases with washing^20^. Aggressive washing can cause the loss of pigment particles from the fabric; therefore, photochromic prints should not be subject to frequent aggressive washing. In addition, the use of a high volume of silica led to an increase in water absorption owing to its reactivity. A large amount of water was absorbed into the silica pores, and a reduction in color was thus observed.

## Conclusions

Bagasse ash has the potential to be a source for amorphous silica extraction using a process with a low concentration of alkaline. This method provides low chemical and energy consumption compared to the traditional method of silica production from molten quartz sand. Acid pre-treatment is recommended to remove impurities in the ash. The silica obtained was light gray in color owing to a high carbon content from incomplete burning. To investigate potential applications of bagasse silica, its use as an extender in a photochromic pigment was studied. Results revealed that silica could be blended at a silica-to-pigment mass ratio of up to 1:10, which is still suitable for thick-layer paining. However, a ratio of less than 1:3 is recommended for thin-layer screen-printing on fabrics. The pigment blends showed very good color fastness when washing; however, frequent aggressive washing is not recommended for photochromic prints.

## References

[1] Department of Alternative Energy Development and Efficiency, Thailand, Thailand alternative energy situation, Bangkok: Ministry of Energy 57p (2017).

[2] Norsuraya S, Fazlena H, Norhasyimi R (2016). Sugarcane bagasse as a renewable source of silica to synthesize Santa Barbara Amorphous-15 (SBA-15). Procedia Eng..

[3] Cordeiro GC, Toledo Filho RD, Tavares LM, Fairbairn EDMR (2009). Ultrafine grinding of sugar cane bagasse ash for application as pozzolanic admixture in concrete. Cem. Concr. Res..

[4] Vassilev SV, Baxter D, Andersen LK, Vassileva CG (2013). An overview of the composition and application of biomass ash: part 2. Potential utilisation, technological and ecological advantages and challenges. Fuel.

[5] Aukkadet R, Tangchirapat W, Jaturapitakkul C (2015). Strength, chloride resistance, and expansion of concretes containing ground bagasse ash. Constr. Build. Mater..

[6] Montakarntiwong K, Chusilp N, Tangchirapat W, Jaturapitakkul C (2013). Strength and heat evolution of concretes containing bagasse ash from thermal power plants in sugar industry. Mater. Des..

[7] Iller, R.K. The chemistry of silica, John Wiley and Sons, New York (1979).

[8] Kalapathy U, Proctor A, Shultz JA (2000). Simple method for production of pure silica from rice hull ash. Bioresour. Technol..

[9] Koner S, Pal A, Adak A (2014). Application of silica gel factory waste for methyl orange dye removal. Int. J. Environ. Waste Manage..

[10] Affandi S, Setyawan H, Winardi S, Purwanto A, Balgis R (2009). A facile method for production of high-purity silica xerogels from bagasse ash. Adv. Powder Technol..

[11] Alves, R. H., Reis, T. V. S., Rovani, S. & Fungaro, D. A. Green synthesis and characterization of biosilica produced from sugarcane waste ash. J. Chem. 2017. article ID 6129035 (2017).

[12] Amin A, Khattak S, Noor S, Ferroze I (2016). Synthesis and characterization of silica from bottom ash of sugar industry. J. Cleaner Prod..

[13] Rovani S, Santos JJ, Corio P, Fungaro DA (2018). Highly pure silica nanoparticles with high adsorption capacity obtained from sugarcane waste ash. ACS Omega..

[14] Thakur, V. K. & Kessler, M. R. Green biorenewble biocomposites: From knowledge to industrial applications, Apple academic press, Oakville (2015).

[15] International Organization for Standardization. ISO 105 C10-06: Textile – Test for color fastness – Part 10: Color fastness to washing with soap or soap and soda (test no. A), (2006).

[16] Williams, E. Global Production Chains and Sustainability: The case of high-purity silicon and itsapplications in IT and renewable energy. United Nations University/Institute of Advanced Studies, (2000).

[17] Bergna, H.E. Colloid chemistry of silica. In H.E Bergna and W.O. Roberts (Ed.), Colloidal silica: Fundamentals and applications (pp. 9-35). Boca Raton, FL: CRC Press (2006).

[18] Zhuravlev LT (1993). Surface characterization of amorphous silica—a review of work from the former USSR. Colloids Surf., A..

[19] Krysztafkiewiez A, Binkowski S, Jesionowski T (2002). Adsorption of dyes on a silica surface. Appl. Surf. Sci..

[20] Chowdhury MA, Joshi M, Butola BS (2014). Photochromic and thermochromic colorants in textile applications. J. Eng. Fibers Fabr..

